# Generativity and Gendered Pathways to Health: The Role of Human, Social, and Financial Capital Past Mid-Life

**DOI:** 10.3390/ijerph19094956

**Published:** 2022-04-19

**Authors:** Yu-Chih Chen, Natalee Hung, Bobo H. P. Lau, Rebecca M. P. Choy Yung, Ellmon S. M. Fung, Cecilia L. W. Chan

**Affiliations:** 1 Department of Social Work and Social Administration, The University of Hong Kong, Pok Fu Lam, Hong Kong SAR, China; nataleeh@hku.hk (N.H.); cecichan@hku.hk (C.L.W.C.); 2 Social Policy Institute, Washington University in St. Louis, St. Louis, MO 63045, USA; 3 Department of Counselling and Psychology, Hong Kong Shue Yan University, North Point, Hong Kong SAR, China; hplau@hksyu.edu; 4 Golden Age Foundation, Wan Chai, Hong Kong SAR, China; rebecca@goldenage.foundation (R.M.P.C.Y.); ellmon.fung@gmail.com (E.S.M.F.)

**Keywords:** generativity, gender difference, physical health, mental well-being, mid-life, older adults

## Abstract

Generativity has recently received increasing attention as a key contributor to healthy aging. Personal resources and social expectations are shown to influence the desire to be generative and that generativity affects later-life health. However, whether generativity has a mediating role in linking its driving factors and health, and how gender may affect these pathways, is underexplored. Cross-sectional online data from 1085 Hong Kong residents aged 45+ were collected between November and December 2020. Latent variable path analysis was used to examine the mediating effect of generativity between human, social, and financial capital, and physical and mental well-being. Gendered pathways were investigated using multi-group analysis. Results showed that human, social, and financial capital contributed to better health through generativity, and gendered pathways were observed. Human capital had a stronger effect on generativity for men, but for women, social capital was vital for increased generativity and consequently improved health. Findings suggested that health benefits of generativity depend on different capital drivers and differ by gender. Implications for program development that aim to facilitate health should include generativity components that maximize physical and psychosocial engagement so that individuals can reap the health benefits through contributions to others.

## 1. Introduction

Generativity, a concern for and behaviors that aim to contribute to the welfare of others [1,2], is integral to the healthy process of aging [3]. Examples of generativity are theoretically classified into four distinct types: *biological* (e.g., bearing and nursing children or grandchildren), *parental* (e.g., offering material or emotional support for younger generations; passing down family traditions), *technical* (e.g., teaching and developing skills and knowledge), and *cultural* (e.g., passing meaning or values to next generations) [4]. Although generativity is thought to peak in mid-life when generative roles (e.g., caregiver, volunteer, mentor) and resources (e.g., human, social, and financial) are greatest, research has shown that it can remain high in later life, even if older adults experience a reduction in their social roles due to life-transition events, such as retirement or family dissolution [5,6]. While generativity has been increasingly recognized as a critical element of healthy aging, the current evidence on generativity is segmented, either focusing on the correlates of generativity or on its potential impacts. There are no systematical investigations that link generativity with its driving factors and outcomes simultaneously.

An early model of generativity [7] suggested that generativity is not only motivated by individuals’ psychological desire (“*inner desire*” the need to be needed) but is also shaped by their social context (“*cultural demand*” such as opportunities and cultural expectations). This implies that *heterogeneity* in the development of generative concern and behavior can be expected for individuals, depending on their resources and the social contexts in which they are situated. Recent revisions to this model of generativity [8] proposed that rather than focusing exclusively on its motivators, the model should be extended to establish the theoretical connection between generativity and well-being outcomes. This extension led to a complete picture of the generativity model, where generativity, driven by both inner desire and cultural demands, also affects health and well-being in mid- and later-life. However, as individuals are shaped by their varied roles, expectations, and experiences over the life course, there are various pathways through which generativity, its antecedents, and outcomes are linked.

### 1.1. Capital Drivers of Generativity

A theoretical framework which previous studies have used to examine the driving factors of generativity is the capital perspective, where generativity is conceptualized as a stock that can be increased through the input of different forms of capital [9,10,11]. Capital types such as financial (e.g., income, assets or financial satisfaction), human (e.g., education, attainment or good health), and social (e.g., volunteering, community or organizational engagement, social contact or network) capital and their effects on generativity have been tested, and all showed positive associations with generativity [9]. Human capital can be defined as productive qualities that cannot be separated from the individual [12], such as knowledge and skills, and is often captured by educational attainment or training [10]. Social capital can be defined as features of social structures that “*facilitate certain actions of individuals who are within the structure*” [13], and has been represented by items such as group membership [14], social integration, and the establishment of social networks [10]. Financial capital can be broadly defined as characteristics that reflect an individual’s financial well-being [15], including the ability to effectively manage economic resources [16].

Human, social, and financial capital have been observed to have independent effects on generativity. Higher levels of education, as a dimension of human capital and an indication of how much knowledge and skills one may be able to pass on to others, were beneficial to generativity [9]. This was confirmed in a separate study which found that *lack* of education incurred feelings of inadequacy, which consequently limited generative expression [17]. Similar relationships were observed for social capital. Having diverse social networks, as well as feeling socially connected, were integral building blocks of generativity [9,17]. Engaging in civic and productive activities, such as volunteering, caregiving or political engagement, was also associated with greater generativity [18,19], although the potential for heterogeneous effects between different groups must also be considered. A previous study showed that only certain types of volunteering had an effect on generativity depending on whether the respondent was retired, and this was hypothesized to be driven by the different cultural expectations placed on individuals based on retirement status: engagement in knowledge-based volunteering was associated with greater generativity among retired respondents, while among working respondents, this association was observed for the activity of caring for vulnerable groups [9]. Evidence has also shown that financial limitations, such as lower financial capability or lack of resources, may inhibit an individual’s self-perceived ability or willingness to contribute to the next generation or restrict opportunities to engage in generative activities [20]. Overall, understanding generativity as a resource that can be grown through investment in its capital drivers is an important theoretical underpinning for the exploration of its effects on well-being outcomes.

### 1.2. Impacts of Generativity

An increasing body of research has linked generativity with a range of desirable health outcomes, though most studies tend to examine a single dimension of well-being outcomes (e.g., physical or mental health) at a time. Evidence has suggested that generative concern is significantly associated with physical health, including lower odds of mobility limitations, onset of disability, mortality, and higher activity functioning [5,21]. Generativity has also been associated with mental well-being, including positive affect, self-worth, social connectedness, greater life satisfaction [19,22,23], lower depressive symptoms, negative affect, and loneliness [24]. Additionally, generativity was found to promote a sense of purpose or satisfaction from contributing to others [2].

The mechanisms that link generativity with better well-being outcomes can be theoretically framed by the socioemotional selectivity theory, in that middle-aged and older adults may prioritize engagements or activities they consider *meaningful* due to a perceived limited time horizon [25]. In other words, the socioemotional selectivity theory suggests that individuals may spend time on activities that they thought to be purposeful to make their remaining lifetime both valuable and meaningful. Therefore, individuals may have the tendency or desire to offer care, pass down their knowledge or skills, or contribute to others through engaging in varied generative activities (e.g., volunteer sessions, community activities or intergenerational programs). These generative activities or actions that individuals purposively engage in may have health implications through two mechanisms: (1) through the positive thoughts and emotions that flow from generative self-perceptions [2], leading to improved mental well-being, or (2) through participation in activities that involve physical, social, and cognitive engagement that subsequently maintain health functioning [26,27]. For example, intergenerational programs offer varied activities to connect middle-aged and older adults with children in need. Program participants could exercise varied health functions through sport, emotional support, and teaching and reading, which contribute to their physical, mental, and cognitive health. In sum, there is empirical support for mental and behavioral pathways that link generativity to improved mid- and later-life well-being.

### 1.3. Gendered Pathway to Generativity

Although most models of generativity suggest heterogeneity in the development of generative concerns due to varying cultural demands such as opportunities or norms embedded in a society, few studies have taken sociocultural expectations into consideration. There have been calls for more studies to explore the role of social context in the realization of generativity, given the influence of social and cultural environments in dictating normative expectations and opportunities for generativity, its driving factors, and outcomes [18]. Among all the sociocultural influences in the development of generativity, gender differences are particularly salient because of the differential expectations and norms society places on men and women in their formative years, which eventually lead to variations in their expression of generativity [28]. Although gender norms evolve over time, studies, i.e., [29,30] have generally supported the notions that women, as girls, are more likely to receive training in areas related to family welfare (e.g., offer care to family members) or are expected to have a nurturing role. In contrast, the socialization process for men is often associated with the roles related to provision (e.g., to offer material assistance) or accomplishments that will bring impacts (e.g., teaching). These varied socialization experiences contribute to the shaping of gendered self-perceptions and behaviors, which can later manifest in the way men and women express generativity. For example, the feeling of being needed was found to be stronger for men in their identification with the role of a provider that facilitates productivity, often associated with teaching, passing on knowledge, and transferring skills [31]. By contrast, women, with values of close social connectedness and sensitivity to other’s needs [32], tend to support social institutions and other people in activities such as caregiving or volunteering. Consequently, as pathways to generativity from its motivating drivers may differ across gender, differential impacts on well-being outcomes may also be expected. However, the current evidence lacks a direct comparison by gender in examining these pathways. Some studies, for example, either solely focus on women (in examining the association between generativity and mental support and loneliness) [32] or simply examine gender effects with other confounding factors such as parenting status, whether they have children, or human agency [22,23]. Such approaches may ignore the distinct socialization process shaped by the gendered structures and demands, thus obscuring the understanding of how generativity may affect health and how that differs by gender contexts.

### 1.4. The Present Study

Despite a growing number of studies that have examined the correlates and impacts of generativity, these findings have not been fully connected to investigate the mediating role of generativity in these pathways, and they also lack the consideration of the influences of gendered sociocultural expectations. Guided by an extended generativity model, this theoretically-driven study aimed to simultaneously link generativity, its driving factors, and influenced outcomes. Using structural equation modeling, human, social, and financial capital antecedents of generativity and physical and mental health outcomes were studied, with explorations of potential gender differences in these pathways. This study hypothesized that generativity would mediate the relationship between its drivers and health outcomes, and these pathways would differ between men and women due to gendered social roles and expectations.

## 2. Materials and Methods

### 2.1. Data and Sample

The data used were from a large survey conducted by a nonprofit advocacy organization, Golden Age Foundation, between November and December 2020. The survey aimed to understand health-related attitudes and activity engagement among mid-life and older adults during the pandemic. Individuals aged 45 and older, able to read Chinese, and who were living in Hong Kong were eligible for this survey. Participants were required to confirm that they met the study inclusion criteria and provide informed consent before taking the survey. Participants were recruited through Google Forms due to an increase in COVID-19 cases during the data collection period, and internet-based surveys were judged to be a flexible method to gather information from the “young-old” represented in this study [33]. A total of 1109 participants completed the survey, and 24 observations had missing data. Sensitivity tests showed no significant differences in sample demographics except for gender (see Supplementary Table S1). Listwise deletion was therefore employed, and gender was used as a stratified variable in the analyses (*n* = 1085).

### 2.2. Measures

#### 2.2.1. Human, Social, and Financial Capital

Human capital was measured by two observed variables: educational attainment (1 = *college or above*, 0 = *below college*) and engagement in continuing education (e.g., pursuit of degrees, seminars or professional workshops) (1 = *yes*, 0 = *no*) followed the definition documented in the prior literature section. Social capital, defined as social structure, group membership, and social network in the literature, was measured by six observed variables, with four binary variables related to social, organizational, and civic engagement, including whether (1) engaging in community services, (2) participation in educational, cultural, sports or professional associations, (3) voting behavior, and (4) whether providing care to vulnerable groups (1 = *yes*, 0 = *no*). In addition, two variables of social network were used, with one on the frequency of contact with friends or family (0 = *none* to 4 = *every day*) and another on the six-item Lubben Social Network Scale [34] (*range* = 0–30; *α* = 0.91). Lastly, financial capital, defined as the ability to effectively manage economic resources [16], was constructed using the five-item Financial Situation Scale (FSS) [35]. This scale captured participants’ financial capacity by evaluating satisfaction toward dealing with the following financial issues, including savings, debt, household’s current financial situation, ability to meet long-term financial goals, ability to deal with financial crises, and financial management abilities (0 = *very dissatisfied* to 4 = *very satisfied*). This measure had higher internal reliability (*α* = 0.91), and higher scores indicated greater satisfaction on financial management ability. Based on the financial capital definition set in the literature, the FSS—the ability related to financial management—has a better utility to measure financial capability than income and assets. Therefore, this study used FSS as a measure for financial capital, and income and assets were controlled as covariates. All capital factors were modeled as latent factors.

#### 2.2.2. Generativity

The latent variable for generativity was constructed using a short-form Loyola Generativity Scale (LGS), which has been similarly used in prior studies [5,24] and is a suitable instrument for measuring generativity in mid-life and later life [36]. Such a measure has been validated with good psychometric properties among Chinese middle-aged and older populations [18]. Examples of items on this measure include: “*I feel like other people need me*”, “*I try to pass along the knowledge I have gained through my experiences to others*”, and “*I feel as though I have made a difference to many people*”. All items were measured using a four-point Likert scale (0 = *strongly disagree* to 3 = *strongly agree*), and higher scores indicated stronger generativity. Sufficient reliability was found in this study (*α* = 0.87).

#### 2.2.3. Physical Health

The latent variable for physical health was comprised of two observed health variables measured both subjectively and objectively. Self-rated health, used as a reference indicator to reflect better latent physical health, was assessed with a single item rating participants’ current health status (*range* = 1–10), such that higher scores indicated better health. Functional health was measured by participants’ ability to move, walk, and exercise (1 = *fully capable* to 5 = *completely incapable*).

#### 2.2.4. Mental Health

The latent variable for mental health was measured by the five-item World Health Organization Well-being Index (WHO-5), a widely used tool with satisfactory validity and sensitivity to assess subjective psychological health across different populations, including mid-life and older adults [37]. Participants were asked to rate their feelings in the past two weeks on the following sample items: “*I have felt cheerful and in good spirits*”, “*I have felt active and vigorous*”, and “*My daily life has been filled with things that interest me*”. This measure represented a positive self-evaluation of one’s life to be both purposeful and meaningful. A six-point Likert scale (0 = *at no time* to 5 = *all the time*) was used, and higher scores indicated better mental well-being (*range* = 0–25). Internal consistency was high in the current study (*α* = 0.96).

#### 2.2.5. Covariates

Several covariates that are known to influence both generativity and health [24,32] were included: age, gender (1 = *women*, 0 = *men*), marital status (1 = *married*, 0 = *non-married*), employment (1 = *working*, 0 = *non-working*), income, assets, an index of ownership of financial tools (e.g., savings account, life insurance, medical insurance, annuity, stock, derivatives, and foreign currency; *range* = 0–7), and an index of chronic illnesses (e.g., high blood pressure, diabetes, and coronary heart disease; *range* = 0–7).

### 2.3. Analyses

Descriptive statistics, including bivariate associations, were calculated to examine how each variable varied by gender. Path analyses and confirmatory factor analyses (CFAs) were performed using Mplus version 7.4 and were conducted with the weighted least square estimation (WLSMV) to account for the ordinal nature of the variables. To examine the role of generativity in the link between its capital drivers and health, standardized estimates for direct and indirect effects were assessed using significance testing, and bias-corrected standard error bootstrapping with 5000 resamplings was employed to test the mediation pathways. We then calculated the shares of the indirect effects over the total effects to understand the relative importance of mediation effects. The analyses began with whole sample estimates, followed by multi-group estimates stratified by gender. Such an approach has been widely used to examine subgroup differences [24]. Model fit was evaluated using established criteria [38]: comparative fit index (CFI) ≥ 0.90, Tucker–Lewis index (TLI) ≥ 0.90, and root mean square error of approximation (RMSEA) ≤ 0.06. 

## 3. Results

Table 1 displays the sample characteristics and bivariate associations for the study variables across gender. Approximately 60% of participants were women, married, and had a college degree. One-third of participants were aged 45 to 55, and approximately four out of ten remained employed. Participants had an average of two or more financial tools. Gender differences were observed in the indicators of human and social capital, as well as generativity. Compared to men, women were more likely to engage in continuing education, have frequent social contact and larger social networks, and score higher on generativity. Women were also observed to have fewer chronic diseases, possibly because they were, on average, younger; they also had more financial tools than men. The correlation of latent variables (see Supplementary Table S2) showed that generativity had a positive and moderate association with human, social, and financial capital (*r* = 0.41–0.63) as well as physical and mental health (*r* = 0.52–0.54), and the CFA model indicated a reasonable model fit (*χ*^2^_(284)_ = 2525.76, *p* < 0.001, *CFI* = 0.98, *TLI* = 0.98, *RMSEA* = 0.08).

### 3.1. Path Analyses

**Figure 1:**
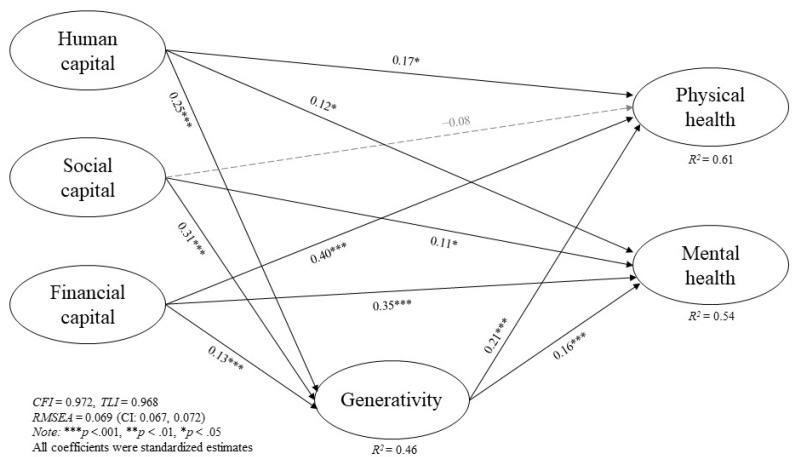
Effects of human, social, and financial capital on physical and mental health, mediated by generativity, total sample. Covariates were controlled for age, gender, marital status, working status, income, assets, and asset ownership. Structural standardized estimates were reported from the final structural models. Black solid lines refer to statistically significant paths; gray dashed lines refer to nonsignificant paths. *** *p* < 0.001, ** *p* < 0.01, * *p* < 0.05.

Figure 1 and the first column of Table 2 show the path model for the overall sample. The full structural model adjusted for covariates demonstrated a good model fit (*χ*^2^_(491)_ = 3060.63, *p* < 0.001, *CFI* = 0.97, *TLI* = 0.97, *RMSEA* = 0.07). The standardized path estimates showed that human (*β* = 0.12–0.17, *p* < 0.05) and financial (*β* = 0.35–0.40, *p* < 0.001) capital were directly related to both physical and mental health, but social capital was only associated with mental health (*β* = 0.11, *p* < 0.05). Human, social, and financial capital were positively correlated with generativity, but the effect of social capital was stronger. Additionally, greater generativity was associated with better physical and mental health, and the effect was strongest for physical health. The effects of covariates on latent constructs of generativity and health outcomes were presented in Supplementary Table S3.

As hypothesized and indicated in Table 2, generativity mediated the pathways from capital drivers to well-being. The significant indirect effects showed that human (*β* = 0.04–0.05, *p* < 0.01), social (*β* = 0.05–0.07, *p* < 0.001), and financial (*β* = 0.02–0.03, *p* < 0.05) capital is associated with generativity, which led to better physical and mental health. Particularly, a full mediation effect of generativity on the associations between social capital and physical health was observed. Having greater human and social capital might lead to better physical and mental health via generativity as the indirect effect explained a moderate proportion of the total effect (20–30%) on the pathways. Such effects were larger than the effect of financial capital, as it only explained a small proportion of the total effect (5–7%) on the pathways to physical and mental health. 

### 3.2. Gendered Differences

**Figure 2:**
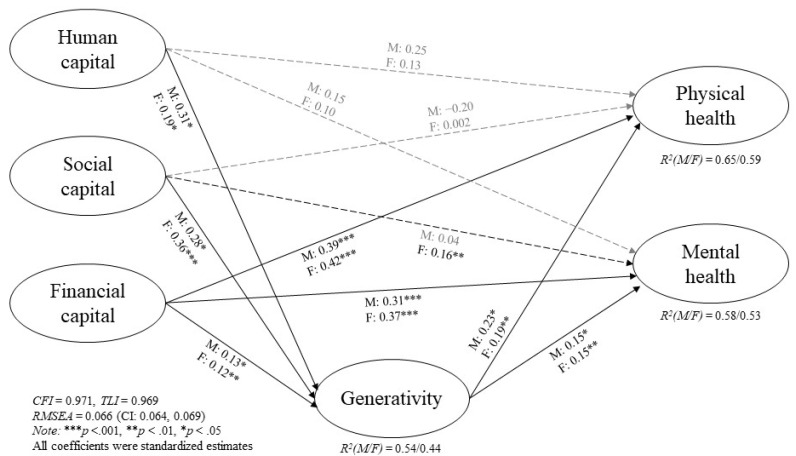
Gender variations in the effects of human, social, and financial capital on physical and mental health, mediated by generativity. Covariates were controlled for age, marital status, working status, income, assets, and asset ownership. Structural standardized estimates were reported from the final structural models. Black solid lines refer to statistically significant paths; black dashed line refers to gender-specific significant paths; gray dashed lines refer to nonsignificant paths. *** *p* < 0.001,** *p* < 0.01, * *p* < 0.05.

Figure 2 and Table 2 present the pathways of generativity that connect capital and health, stratified by gender. The full multi-group structural model yielded a good model fit (*χ*^2^_(1002)_ = 3378.91, *p* < 0.001, *CFI* = 0.97, *TLI* = 0.97, *RMSEA* = 0.07). Similar to the findings for the overall sample, human, social, and financial capital were positively related to generativity for both men and women, although the magnitude of effect differed. As shown in Figure 2, human capital had a stronger impact on generativity for men (*β* = 0.31, *p* < 0.05), but for women, it was social capital that was vital for increased generativity (*β* = 0.36, *p* < 0.001). Greater generativity was also associated with better outcomes, but higher levels of generativity led to comparatively better physical than mental health for both men and women. The strengths of the mediating effect of generativity also differed by gender, depending on the capital driver of the pathway, as shown in Table 2. Among men, the indirect effect explained a moderate proportion of the effects (22–25%) connecting human capital and physical and mental health via generativity. For women, generativity explained a greater proportion of the total effects on physical and mental health through social capital pathways. This was particularly pertinent for physical health, as the effect of social capital on physical health was only observed when mediated by generativity, and direct effects were not significant. The effect of financial capital on these pathways to health was less apparent than human and social capital. 

## 4. Discussion

This study is among the first to systematically examine the role of generativity in the connection between its capital drivers and health, with further explorations of gendered pathways. Results showed that, first, human, social, and financial capital are important factors of generativity, but social capital demonstrated a comparatively stronger and facilitating effect on generativity. Additionally, generativity was significantly associated with better physical and mental well-being. Taken together, generativity was found to mediate the pathways between social, human, and financial capital and health. Second, this study hypothesized and demonstrated that, overall, all capital drivers contributed to better health, but there were also distinct gendered pathways to health through generativity. Results of multigroup analyses showed that men fared better in health through contributions to others if they possessed higher levels of human capital, but for women, this pathway was primarily driven by social capital. 

The mediating role of generativity revealed in this study confirms that generativity is motivated by human, social, and financial capital drivers, and that it subsequently affects physical and mental health [8]. The finding that capital drivers are closely related to generativity is consistent with prior research [14,18]. The positive association of human capital with generativity is likely because human capital—measured by educational attainment and engagement in continuing education—supports the transmission of knowledge and experience, and represents a lifelong resource that strengthens individuals’ capacities to contribute to others and make meaningful connections [9]. A previous study had shown that feelings of inadequacy due to a widening educational gap between themselves and the younger generation made older adults reluctant to realize their generative intents, leading to more passive expressions of generativity (e.g., waiting for an invitation to offer help) [18]. The substantial impact of social capital (e.g., volunteering, voting, and social connection) on generativity is also largely supported in the literature. Middle-aged and older adults often make contributions through productive, civic, and social activities occurring in their family, friend, or community spheres [39]. In these opportunities for generative involvement, individuals may feel more useful and respected, further promoting the desire to help others [40]. While an association between financial capital and generativity was observed in our study, its effect was less pronounced compared to the other capital types, and it was only marginally significant in the gender-specific analyses. These findings echo a previous study that had examined the capital antecedents of generativity, where the effects of human and social capital on generativity were also stronger and more consistent, compared to financial capital [9].

Our study showed that those with higher levels of generativity have better physical health and mental well-being. While previous studies which predominantly focused on one well-being outcome at a time also provided evidence for a positive link between generativity and health [5,32], our study offers a “whole-person” view that captures multiple aspects of well-being. The health benefits can be partly explained by the socioemotional selectivity theory: as they reflect on what it means to live a meaningful life, middle-aged and older adults may be spurred to adopt generative roles and behaviors as a basis for a positive sense of identity [23]. Another theoretical explanation can be framed by the social model of health promotion [27], as highly generative individuals, motivated by their human and social capital, tend to occupy multiple roles (e.g., helper, teacher, volunteer or mentor) that may involve physical, mental, and social engagement, which then leads to improved health status [26]. Collectively, these theoretical frameworks call for more studies to explore how psychological and behavioral mechanisms may operate in the associations between generativity and health outcomes [21,31], which this study was unable to examine as these data were not captured in the survey. 

This study also highlighted gendered pathways in the mediating role of generativity between its capital drivers and health. Although both human and social capital related to generativity, which then led to better health, these pathways operated differently between men and women. For men, the health benefits of generativity were predominantly influenced by human capital, but among women, it was social capital that facilitated the health benefits through generativity. Such findings support the theoretical underpinning that pathways to health are shaped by sociocultural expectations [28,30], and these vary by gender. As men tend to make contributions in areas related to material-provision or skill-transfer whereas women tend to be generative via activities such as caregiving and offering emotional support [41], different capital drivers may facilitate generativity, leading to differential health consequences between men and women. Previous studies had explored the moderating effect of gender through the use of interaction terms, and while they had concluded that there was a lack of evidence for the gendered effects of generativity [22,23], the motivators of generativity were not considered in their analyses. However, by stratifying the analyses by gender, the present study was able to offer a clearer picture of how contributions to others (i.e., capital drivers to generativity) and contributions to oneself (i.e., generativity to outcomes) can be influenced by the gendered context.

This study has some limitations. First, although the LGS is a widely used scale with satisfactory validity, it may not capture all aspects of generativity. The generativity theory conceptualizes generativity to have multiple distinct forms, including concern, commitment, and behavior [7], but this scale predominantly taps into the notion of generative concern. Future studies should consider examining other aspects of generativity, such as commitment or behavior, in its pathways to health. Second, the interpretation of the findings is affected by cross-sectional data collection and the COVID-19 pandemic context. The theoretical framework that guided this study hypothesized that investment in capital increases generativity, which consequently leads to better health. However, those with greater physical and mental health could likewise be socially engaged in ways that lead to higher generativity. For example, prior research has shown that perceived physical health was positively correlated with generativity concerns and generativity behavior that was measured by volunteering [10]. However, chronic conditions and depressive symptoms were not associated with generativity [9]. Additionally, as the data were collected when there were increased incidence of COVID-19 cases in Hong Kong, the COVID-19 pandemic may have also affected these tested associations. Social distancing measures, such as lockdowns, work-from-home arrangements, or social gathering bans, significantly impacted individuals’ health and opportunities for engagement [42], and these circumstances also reflect the limitations of our cross-sectional design. For example, social distancing restriction impedes social interaction and, based on this study, lack of opportunities for meaningful and quality connections would lead to lower generativity, consequently resulting in poor physical and mental health [43]. Conversely, physical inactivity and loneliness due to social distancing also compromises physical and mental health [44], resulting in reduced concerns for contribution and opportunity for social engagement. The temporality of this dynamic relationship that is intertwined with the COVID-19 pandemic cannot be delineated due to the contemporaneous measurement design. Longitudinal studies would be beneficial to capturing directionality in the causal mechanisms that connect generativity, motivating factors, and health embedded in different stages of pandemic situations. Lastly, the sample contains relatively high-functioning participants who are likely to be computer literate, willing to participate in voluntary research, and with higher educational attainment. It is possible that self-selection bias may have occurred in this study, limiting the generalizability of the findings.

The results of this study have implications for programs, research, and agencies to further develop generativity. As generativity is a key element for healthy aging [3], programs should be purposively designed in ways to promote generativity through physical and psychosocial engagement [26]. Activities that contribute to others with physical and psychosocial components (e.g., volunteering or mentoring) could help individuals to reap health benefits through engaging in these activities. Additionally, the programs should consider strategies for maintaining and developing generativity tailored to the local cultural contexts. For example, a qualitative study in Hong Kong showed that older adults strived to be “less of a burden” (e.g., by taking good care of themselves or helping with household chores) or through mentorships (e.g., teaching younger generations moral and behavioral codes) as a realization of generativity [17]. A culturally-sensitive approach to understanding generativity is essential to building and developing strategies for harnessing generativity to achieve healthy aging. Additionally, the positive generativity-health nexus suggests that generativity can be modified to improve health [45]. However, high functioning individuals, such as those observed in our study, may be more likely to engage in generative activities, impeding a comprehensive assessment of the association between generativity and health. More research and intervention trials that include individuals from diverse conditions, like the high-intensity Experience Corp. program (e.g., engaging in generative activities such as teaching and volunteering) that recruits physical-active individuals [45], or a sedentary-based intervention (e.g., writing a reflective journal on the generative concerns when offering help) that involves much older and frail adults [32], should be conducted to unfold the health utility of generativity. Furthermore, as the manifestation of generativity depends primarily on its capital drivers and varies by gender, the program developers should consider gender differences in generativity that are affected by varied types of capital drivers. Programs that facilitate educational and social engagement can fulfill the generative desires of middle-aged and older men and women, create opportunities and expand capacities for generative actions, and also serve as a potential pool for agencies seeking volunteers by identifying individuals with higher generativity, particularly among those with higher educational attainment and social networks [9].

## 5. Conclusions

By demonstrating the mediating effect of generativity between its capital drivers and health outcomes, as well as the stratified pathways by gender, this study has shown that improvements in health can be achieved through investment in the capital drivers of generativity, and that gendered sociocultural expectations have a role to play for how generativity, and consequently, health, can be most effectively promoted. Understanding both are important perspectives to integrate into program development which aims to support multidimensional health in later life.

## Figures and Tables

**Table 1:** Sample characteristics and bivariate analyses by gender.

Variables	Whole	Men	Women	*χ*^2^ or *t*
	M (SD) or %	M (SD) or %	M (SD) or %	
Human capital (HC)				
Education attainment (*Above college*)	57.53%	58.49%	56.91%	*χ*^2^ = 0.27
Continuing education (*yes*)	57.08%	48.62%	62.56%	*χ*^2^ = 20.96 ***
Social capital (SC)				
Engaged in community services (*yes*)	63.93%	60.78%	65.97%	*χ*^2^ = 3.09
Engaged in educational, cultural or professional association (*yes*)	57.08%	55.50%	58.10%	*χ*^2^ = 0.73
Whether voted (*yes*)	85.57%	84.86%	86.03%	*χ*^2^ = 0.29
Providing care for vulnerable groups (*yes*)	70.87%	69.95%	71.47%	*χ*^2^ = 0.29
Frequency of social contact with friends or families ^‡^	1.94 (0.97)	1.79 (1.00)	2.05 (0.93)	*t* = −4.42 ***
Social network (*Lubben social network scale*)	12.63 (6.45)	11.25 (6.29)	13.52 (6.40)	*t* = −5.80 ***
Financial capital (FC, *Financial situation scale*)	12.57 (4.48)	12.49 (4.63)	12.62 (4.38)	*t* = −0.47
Generativity (GEN, *Loyola generativity scale*)	8.19 (2.88)	7.72 (2.95)	8.50 (2.82)	*t* = −4.41 ***
Physical health (PH)				
Self-rated health	6.79 (1.94)	6.50 (1.97)	6.87 (1.91)	*t* = −1.88
Mobility limitation ^†^	1.27 (0.60)	1.29 (0.66)	1.25 (0.56)	*t* = 0.95
Mental health (MH, *WHO-5*)	11.96 (5.44)	11.61 (5.47)	12.19 (5.42)	*t* = −1.72
Covariates				
Gender (*woman*)	60.69%	--	--	--
Age (*age 45–55*)	29.58%	23.85%	33.28%	*χ*^2^ = 16.36 ***
55–65	46.98%	47.48%	46.66%	
65+	23.44%	28.67%	20.06%	
Marital status (*married*)	60.60%	70.87%	53.94%	*χ*^2^ = 31.78 ***
Employment (*working*)	44.63%	44.27%	44.87%	*χ*^2^ = 0.04
Income ^‡^	2.59 (1.49)	2.68 (1.53)	2.54 (1.46)	*t* = 1.58
Assets ^‡^	2.74 (1.29)	2.70 (1.32)	2.76 (1.28)	*t* = −0.80
Financial ownerships	2.30 (1.65)	2.04 (1.67)	2.47 (1.62)	*t* = −4.14 ***
Number of chronic diseases ^†^	0.75 (0.93)	0.92 (0.98)	0.63 (0.87)	*t* = 4.99 ***

Notes: ^†^ indicates unequal variance adjustment for the *t* tests. ^‡^ indicates ordinal measures. *** *p* < 0.001,** *p* < 0.01, * *p* < 0.05.

**Table 2:** Significance of standardized paths and relative importance of indirect paths.

Paths	Total Sample	Men	Women
Direct effect	*β*	*β*	*β*
HC → PH	0.17 *	0.25 *	0.12
HC → MH	0.11 *	0.15 *	0.1
SC → PH	−0.08	−0.20	0.002
SC → MH	0.11 *	0.04	0.16 **
FC → PH	0.40 ***	0.39 ***	0.42 ***
FC → MH	0.35 ***	0.31 ***	0.37 ***
Indirect effect			
HC → GEN → PH	0.05 **	0.07 *	0.04 ^†^
	(*CI*: 0.03, 0.10)	(*CI*: 0.03, 0.22)	(*CI*: 0.00, 0.09)
HC → GEN → MH	0.04 **	0.05 *	0.03 ^†^
	(*CI*: 0.02, 0.08)	(*CI*: 0.01, 0.18)	(*CI*: 0.00, 0.07)
SC → GEN → PH	0.07 ***	0.06	0.07 ***
	(*CI*: 0.03, 0.11)	(*CI*: −0.01, 0.14)	(*CI*: 0.02, 0.13)
SC → GEN → MH	0.05 ***	0.04	0.05 **
	(*CI*: 0.02, 0.08)	(*CI*: −0.01, 0.11)	(*CI*: 0.02, 0.10)
FC → GEN → PH	0.03 *	0.03	0.02 ^†^
	(*CI*: 0.01, 0.06)	(*CI*: 0.00, 0.08)	(*CI*: 0.00, 0.06)
FC → GEN → MH	0.02 *	0.02	0.02 ^†^
	(*CI*: 0.01, 0.04)	(*CI*: 0.00, 0.07)	(*CI*: 0.00, 0.04)
Relative importance of indirect path	%	%	%
HC → PH + HC → GEN → PH	100.00 ^‡^	100.00 ^‡^	100
HC → PH	77.27 ^‡^	78.12 ^‡^	75
HC → GEN → PH	22.78 ^‡^	21.88 ^‡^	25
HC → MH + HC → GEN → MH	100.00 ^‡^	100.00 ^‡^	100
HC → MH	73.33 ^‡^	75.00 ^‡^	76.92
HC → GEN → MH	26.67 ^‡^	25.00 ^‡^	23.08
SC → PH + SC → GEN → PH	100	100	100
SC → PH	--	--	0
SC → GEN → PH	--	--	100.00 ^‡^
SC → MH + SC → GEN → MH	100.00 ^‡^	100	100.00 ^‡^
SC → MH	68.75 ^‡^	50	76.19 ^‡^
SC → GEN → MH	31.25 ^‡^	50	23.81 ^‡^
FC → PH + FC → GEN → PH	100.00 ^‡^	100.00 ^‡^	100.00 ^‡^
FC → PH	93.02 ^‡^	92.86 ^‡^	95.45 ^‡^
FC → GEN → PH	6.98 ^‡^	7.14	4.55
FC → MH + FC → GEN → MH	100.00 ^‡^	100.00 ^‡^	100.00 ^‡^
FC → MH	94.59 ^‡^	93.94 ^‡^	94.87 ^‡^
FC → GEN → MH	5.41 ^‡^	6.06	5.13

Notes: *β* = standardized path coefficients. HC = human capital; SC = social capital; FC = financial capital; GEN = generativity; PH = physical health; MH = mental health. ^‡^ indicates the effects of path were significant. *CI* = 95% confidence interval produced from bootstrapping procedures. *** *p* < 0.001, ** *p* < 0.01, * *p* < 0.05, ^†^ *p* < 0.10.

## Data Availability

Data available upon request due to ethical and privacy restrictions. Data are not publicly available due to ethical and privacy restrictions. Data presented in this study are available upon request from the corresponding author.

## Supplementary Materials

The following supporting information can be downloaded at: https://www.mdpi.com/article/10.3390/ijerph19094956/s1, Table S1: Missing data analyses, Table S2: Correlation of latent variables, Table S3: Effect of covariates among total participants.
