# Supplementary material for: Generativity and Gendered Pathways to Health: The Role of Human, Social, and Financial Capital Past Mid-Life
Source: Int J Environ Res Public Health. 2022 Apr 19;19(9):4956. doi: 10.3390/ijerph19094956 (PMC9099985; doi:10.3390/ijerph19094956)
Supplement: Supplementary file 1 [file ijerph-19-04956-s001.zip › ijerph-1662724-supplementary.pdf]

Supplementary Materials

Table S1. Missing data analyses.

Variable	Not missing	Missing	χ^2 or
	n (%)	*n* (%)	*t*-statistic (*df*)
Generativity, *Mean* (*SD*)	8.18 (2.90)	7.91 (2.48)	$t(983) = 0.44$
Age			
45–55	277 (28.79)	5 (21.74)	
56–65	445 (46.26)	15 (65.22)	$\chi^2(2) = 3.41$
>65	240 (24.95)	3 (13.04)	
Gender			
Men	399 (41.48)	3 (13.04)	$\chi^2(1) = 7.52$ **
Women	563 (58.52)	20 (86.96)	
Marital status			
Married	580 (60.29)	14 (60.87)	
Single	256 (26.61)	7 (30.43)	$\chi^2(2) = 0.46$
Divorced or widowed	126 (13.10)	2 (8.70)	
Educational attainment			
Below college	394 (40.96)	10 (43.48)	$\chi^2(1) = 0.06$
College or above	568 (59.04)	13 (56.52)	

*** $p < 0.001$, ** $p < 0.01$, * $p < 0.05$.

Table S2. Correlation of latent variables.

	1	2	3	4	5	6
1. Human capital	--					
2. Social capital	0.64 ***	--				
3. Financial capital	0.51 ***	0.36 ***	--			
4. Generativity	0.63 ***	0.59 ***	0.41 ***	--		
5. Physical health	0.62 ***	0.42 ***	0.70 ***	0.54 ***	--	
6. Mental health	0.59 ***	0.49 ***	0.64 ***	0.52 ***	0.73 ***	--

*** $p < 0.001$, ** $p < 0.01$, * $p < 0.05$.

Table S3. Effect of covariates among total participants.

Variable	Generativity	Physical health	Mental health
	β	β	β
Age (*ref*: age 65+)			
45–55	−0.03	0.06	−0.13 ***
55–65	−0.07	0.06	−0.10 **
Woman (*ref:* man)	0.13 ***	−0.03	0.003
Married (*ref:* not married)	0.06	0.09 **	0.06 *
Work (*ref:* not working)	−0.01	−0.08	−0.05
Income	0.16 ***	0.11 **	0.13 ***
Asset	0.09 **	0.20 ***	0.15 ***
Financial ownerships	0.24 ***	0.21 ***	0.28 ***
Number of chronic diseases	−0.05	−0.22 ***	−0.10 ***

β = standardized path coefficients. *** $p < 0.001$, ** $p < 0.01$, * $p < 0.05$.
